# Protective effects of *Cynara scolymus* leaves extract on metabolic disorders and oxidative stress in alloxan-diabetic rats

**DOI:** 10.1186/s12906-017-1835-8

**Published:** 2017-06-19

**Authors:** Maryem Ben Salem, Rihab Ben Abdallah Kolsi, Raouia Dhouibi, Kamilia Ksouda, Slim Charfi, Mahdi Yaich, Serria Hammami, Zouheir Sahnoun, Khaled Mounir Zeghal, Kamel Jamoussi, Hanen Affes

**Affiliations:** 1 Laboratory of pharmacology, Faculty of Medicine, University of Sfax, Avenue Majida Boulila, 3029 Sfax, Tunisia; 2 Laboratory of Plant Biotechnology, Faculty of Sciences, University of Sfax, Sfax, Tunisia; 3 Laboratory of Anatomopathology, CHU Habib Bourguiba, University of Sfax, Sfax, Tunisia; 4 Biochemistry Laboratory, CHU Hedi Chaker, University of Sfax, Sfax, Tunisia

**Keywords:** Diabetes, Artichoke, Liver, Kidney, Pancreas, HPLC, Antioxidant activity

## Abstract

**Background:**

Diabetes mellitus (DM) is associated with hyperglycemia, inflammatory disorders and abnormal lipid profiles, currently the extracts from leaves of *cynara scolymus* has been discovered to treat metabolic disorders and has been stated by multitudinous scientists according to a good source of polyphenols compounds. The present study aimed to evaluate the protective effect of the ethanol leaves extract of *C. scolymus* in alloxan induced stress oxidant, hepatic-kidney dysfunction and histological changes in liver, kidney and pancreas of different experimental groups of rats.

**Methods:**

We determinate the antioxidant activity by ABTS^**.+**^ and antioxidant total capacity (TAC) of all extracts of *C. scolymus* leaves, the inhibition of α-amylase activity in vitro was also investigated. Forty male Wistar rats were induced to diabetes with a single dose intraperitoneal injection (i.p.) of alloxan (150 mg/kg body weight (b.w.)). Diabetic rats were orally and daily administrated of ethanol extract from *C. scolymus* at two doses (200-400 mg/kg, b.w) or (12 mg/kg, b.w) with anti-diabetic reference drug, Acarbose for one month. Ethanol extract of *C. scolymus* effect was confirmed by biochemical analysis, antioxidant activity and histological study.

**Results:**

The results indicated that the ethanol extract from leaves of *C. scolymus* showed the highest antioxidant activity by ABTS^**.+**^ (499.43g± 39.72 Trolox/g dry extract) and (128.75 ± 8.45 mg VC /g dry extract) for TAC and endowed the powerful inhibition in vitro of α-amylase activity with IC50=72,22 ug/uL. In vivo, the results showed that ethanol extract from the leaves of *C. scolymus* (200-400 mg/kg) decreased significantly (*p* < 0.001) the α-amylase levels in serum of diabetic rats, respectively associated with significant reduction (*p* < 0.001) in blood glucose rate of 42,84% and 37,91% compared to diabetic groups after 28 days of treatment, a significant lowered of plasma total cholesterol (T-Ch) by 18,11% and triglyceride (TG) by 60,47%, significantly and low-density lipoproteins (LDL-C) by 37,77%, compared to diabetic rats, moreover, the administration of ethanol extract appears to exert anti-oxidative activity demonstrated by the increase of CAT, SOD and GSH activities in liver, kidney and pancreas of diabetic rats. This positive effect of the ethanol extract from *C. scolymus* was confirmed by histological study.

**Conclusion:**

These observed strongly suggest that ethanol extract from the leaves of *C. scolymus* has anti-hyperglycemic properties, at least partly mediated by antioxidant and hypolipidemic effects.

## Background

DM is a complicated disease characterized by impaired blood glucose levels resulting from defects in insulin secretion and/or insulin action. Generally, treatment of diabetes focuses on modulating glucose or lipid metabolism or both [1].

The development of diabetic complication is a health problem affecting millions of individuals worldwide. It is a major endocrine disorder affecting nearly 10% of the population all over the world [2]. The global prevalence of diabetes has increased dramatically and is expected to reach 439 million patients by 2030 [3]. Globally, diabetes has shadowed the spread of modern lifestyle and it can be linked to an increase in overweight obesity of population [4].

The development of oxidative stress (OS), underlies the major complications in DM patients [5], OS is an imbalance between oxidants and antioxidants in favor of the former, potentially leading to cell damage and destruction [6]. Several experimental studies have suggested that the increased generation of reactive oxygen species (ROS) causes damage to cells, tissues like pancreas, kidney and liver contributed to diabetic complications [7].

Moreover, dyslipidemia is a common in both insulin deficiency and insulin resistance, which affect enzymes and pathways of lipid metabolism. It is a major risk factor for cardiovascular diseases which is currently a leading cause of morbidity and mortality worldwide. Major metabolic derangements which result from insulin deficiency in DM type 1 are impaired glucose, lipid and protein metabolism [8, 9]. Understanding and improving medication is vital for the management of diabetes and its complications, many of the currently available hypoglycemic drugs for the management of DM have undesired side effects [10]. Hence, in the scientific investigation to find alternative therapeutic strategies for the diabetes treatment has become inevitable. The importance of plants extracts in diabetes management is widely knowledge and a large number of medicinal plants have been documented for their effective role in the diabetes management [11], under hyperglycemic condition various factor such as impairment in insulin mechanism, hyperlipidemia and oxidative stress were causing secondary complication such as diabetes retinopathy, neuropathy and cardiovascular diseases [12]. In recent years, there is growing evidence that plant-foods polyphenols, due to their biological properties, may be unique nutraceuticals and supplementary treatments of dyslipidemia and diabetes [13], being those beneficial effects largely attributed to phenolic compounds, including flavonoids, phenolic acids, lignans and stilbenes on metabolic disorders and complications induced by diabetes [14]. These compounds gained much attention due to their antioxidants activities which potentially have beneficial implications for human health [15]. Human and animal studies showed the potential effects of polyphenols and flavonoids to modulate carbohydrate and lipids metabolisms, decrease glycemia and insulin resistance, increase lipid metabolism and optimize OS [14], moreover various in vitro studies in cultured cells have showed that polyphenols fractions able to enhance glucose uptake into muscle cells and their ability to regulate key enzymes implicated in hepatic glucose output which could decrease glycemia levels [16].

*Cynara scolymus* was an ancient herbaceous perennial plant, originating from the Southern Mediterranean parts of North Africa. Leaves from *Cynara* have been widely used in herbal medicine, since ancient time, as choleretic, bile expelling, hepatoprotective, urinative, antioxidative activities, anti-inflammatory, as well as the ability to inhibit cholesterol biosynthesis [17, 18]. Many studies demonstrated that the phenolic compounds presented mainly in the leaves rather than in *C. scolymus* heads have been documented as the active principles of this plant [15].

Despite long traditional use of *C. scolymus* as medicinal herb, no systemic pharmacological works have been carried out on this potential medicinal plant in Tunisia region. Therefore, the aim of the present study to evaluate for the first investigation the effect of the ethanol extract from the leaves of *C. scolymus* on liver-kidney dysfunction as well as metabolism disorders in alloxan model diabetic rats.

## Methods

### Plant material

Fresh leaves of *Cynara scolymus* were collected during December 2015 from a culture area located in Bizerte region (37°16′28″N 9°52′26″) North West of Tunisia characterize by a humid climate season. The harvested plants were identified in the botany laboratory of the faculty of Siences, Sfax University, Tunisia, by Professor Mohamed Chaeib and a voucher specimen [ACC27] was deposited at the Herbarium of the Laboratory of the Biotechnology Centre of the Technopark of Borj-Cedria. The leaves were manually separated and dried in the shade.

### Preparation of extract

The collected of *C. scolymus* leaves were sliced into smaller pieces and dried at room temperature under shade and then powdered finely and subjected to extraction. Two hundred grams of the powder leaves was fractionated successively with 1 L for (3 Times ×48 h) of following solvents: hexane, ethylacetate, butanol, 75% *v*/*v* (ethanol/H_2_O) and water. All extracts were filtered through Whatman No.1 filter paper in a Buchner funnel. The filtrate was evaporated and removed pressure using a Rotovapor at 40 °C and lyophilized by freeze-dryer (Alpha 1–2 LD plus Martin Christ®) in order to determine the weight and yield of extraction and they were stored at 4 °C until analysis. On the other hand, a second extraction of powdered leaves (500 g) was performed in the same conditions for in vivo study with 75% *v*/*v* (ethanol/H_2_O). After filtration, the ethanol was evaporated under reduced pressure to yield the ethanol extract (30 g).

The choice of extraction solvent was the ethanolic extract from leaves of *C. scolymus* in order to isolate the maximum of all high hydrophilic compounds which responsible for biological activities as compared to other extracts as mentioned.

### Preliminary phytochemical screening for secondary metabolites

Preliminary qualitative phytochemical screening was conducted on all extracts from the leaves of *C. scolymus* using standard procedures [19, 20].

### Determination of total phenolic and flavonoid content

Total phenolic content (TPC) was determined by the Folin-ciocalteau method [21] and expressed as Gallic acid equivalents (GAE)/g dry extract. Total Flavonoid content (TFC) was determined spectrophotometrically using a method based on the formation of a flavonoid aluminum complex [22]. Catechin (CE) was chosen as a standard and TFC was determined in triplicate and expressed in mg (CE)/g dry extract.

### Determination of antioxidant activity of *C. scolymus* leaves extracts

#### Antioxidant capacity by ABTS• + method

The 2,2-azinobis-3-ethylbenzothiazoline-6-sulfonic acid free radical (ABTS^●+^) neutralization was determined using a spectrophotometric, 96-well microplate method described by Re et al. [23].

### Total antioxidant capacities

Total antioxidant capacities (TAC) of extracts were evaluated by phosphomolybdenum method of Prieto et al. [24].

### Identification of phenolic compounds by High-performance chromatographic Liquid analysis (HPLC)

Phenolic compounds analysis of the ethanol extract from the leaves of *C. scolymus* was carried out using an Agilent Technologies 1100 series liquid chromatography (HPLC, Palo Alto, CA) coupled with a UV-vis multiwavelength detector. The separation was carried out on a 250× 8 mm, Eurosphere 100–5, C18 column at ambient temperature. The mobile phase consisted of acetonitrile (solvent A) and water with 0.2% sulphuric acid (solvent B). The flow rate was kept at 0.5 mL min-^1^. The gradient program was as follows: 15% A/85% B, 0–12 min, 40% A/60% B, 12–14 min; 60% A/40% B, 14–18 min; 80% A/20% B, 18–20 min; 90% A/10% B, 20–24 min; and 100% A, 24–28 min. For the preparation of calibration curve, a standard stock solution was prepared in methanol (HPLC grade **≥** 99.9% from Sigma Chemical Company) containing Hydroxytyrosol, Tyrosol, 4HOBenz, Verbascoside, Apigenin-7-Glucoside, Oleuropein, Quercetin, Pinoresinol, Cinnamic acid and Apigenin at the same concentration of 1 g/mL.

The extract was dissolved in 1 mL of methanol making a concentration of 25 mg/mL. Before starting HPLC analysis, all the solutions prepared were filtered with Whatman paper and through a 0.45-μm Millipore filter.

The injection volume was 20 μL and peaks were monitored at 280 nm. Peaks were identified by congruent retention times compared with standards; Hydroxytyrosol, Tyrosol, 4HOBenz, verbascoside, Apigenin-7-Glu, Oleuropein, Quercetin, Pinoresinol, cinnamic acid and Apigenin. Analyses were performed in triplicate.

### Determination of α-amylase inhibition activity by CNPG3 assay

The assay mixture, containing 200 mL of 0.02 M sodium phosphate buffer, 20 mL of a porcine pancreatic α-amylase enzyme and *C. scolymus* leaves extracts in concentration range 25–200 mg/mL, were incubated for 10 min at room temperature followed by addition of 200 mL of starch in all test tubes.

The reaction was terminated with the addition of 400 uL DNS reagent. It was placed in boiling water bath for 5 min, cooled and diluted with 15 mL of distilled water and absorbance was measured at 540 nm. The control sample was prepared without any extract.

The percentage inhibition was calculated according to formula of Nair et al. [25].

$$ \mathrm{Inhibition}\%=\frac{\mathrm{ABS}\ \left(\mathrm{control}\right)-\mathrm{ABS}\left(\mathrm{extract}\right)}{\mathrm{ABS}\ \left(\mathrm{control}\right)}\times 100 $$

IC50 values were determined from plots of percent inhibition versus log inhibitor concentration and were calculated by non linear regression analysis from the mean inhibitory values. Acarbose was used as a specific inhibitor of α-amylase. All tests were performed in triplicate.

### Acute toxicity studies

Acute toxicity study was performed according to the acute toxic classic method. The diabetic rats were divided into seven groups of eight animals each. Group 1 received NACL (0.9%) orally in a volume of 10 mL/kg of body weight (b.w) and served as control. Groups 2, 3, 4, 5, 6 and 7 were administered, respectively, at the doses of 100, 200, 400, 600, 1000 and 2000 mg/kg orally in a volume of 10 mL/kg. After the oral administration of ethanol extract from leaves of *C. scolymus*, the rats were observed continuously for 24 h for behavioral and neurological changes and then at 24 and 72 h for any lethality [26].

### Experimental animals

Forty (*n* = 40) healthy male Wistar albino rats, aged between 10 and 12 weeks and with body weight of 160–200 g obtained from SIPHAT Institute of Tunis.

All rats were fed with a standard laboratory diet composed of corn; soya and vitamins supplied by the company of animal’s nutrition Sfax; Tunisa and water ad libitum. Food was withdrawn 12 h prior and during the experiment.

The experimental protocol of the present study was conducted in accordance with the guide for the care and use of laboratory animals issued by the University of Sfax, Tunisia, and approved by the Committee of Animal Ethics (Protocol no. 94–1939). Before initiation of the experiment, the rats were acclimatized for a period of 7 days standard environmental conditions such as temperature (24 ± 4 °C), relative humidity (45–55%) and a 12 h dark/light cycle were maintained in the quarantine. All animals were randomly assigned to the control and treatment groups, each of which consisted of eight rats.

### Induction of diabetes in experimental animals

Rats were injected with a freshly prepared solution of alloxan monohydrate (i.p.) in saline (300 mM NaCl) at a dose of (150 mg/kg, b.w.) [27]. Alloxan injection can provoke fatal hypoglycemia as a result of reactive massive release of pancreatic insulin, so rats were also given orally 5–10 mL of a 20% glucose solution after 6 h. Rats were then kept for the next 24 h on a 5% glucose solution as beverage to prevent too severe hypoglycemia [28]. After 2 weeks, rats displaying glycosuria and hyperglycemia (over 2 g/L) were chosen for the experiments.

### Experimental design

The animals were randomly divided into five groups (*n* = 8) as follows:

Group 1 and 2 served as a normal and diabetic controls (receiving only the water and the standard diet), Groups 3 and 4, diabetic rats respectively received the 75% ethanol extract from leaves of *C. scolymus* at a dose of (200 mg/kg&400 mg/kg) daily in one mL by oral gavage, Group 5 served as diabetic rats received standard diabetic drug (acarbose, 12 mg/kg per day orally) [16].

### Measurement of blood glucose and body weight

Blood glucose concentration in all experimental groups were recorded following 12 h fasting each day, at 8:00 a.m., before feeding rats, using a glucometer (Bionime, Pharmatec, Tunisia) by glucose oxidase-peroxidase method using glucose test strips.

Rats were weighed individually at weekly intervals using a Mettler Toledo® digital precision balance with a sensitivity of 0.01 g (Model MT-500D), and the body weights were recorded to calculate weekly body weight gains. The fasting body weight and blood glucose levels were estimated on 1, 7, 14, 21 and 28 days, periodically.

### Oral glucose tolerance test (OGTT)

OGTT evaluates the ability to respond appropriately to a glucose challenge [29]. OGTT was conducted in control and treated groups of rats, 24 h before decapitation of rats. All groups were administrated glucose (2 g/kg) by gastric gavages route.

Blood glucose levels were determined at 0, 30, 60, 120 and 240 min subsequently to receive glucose and fasting glucose was measured.

Blood samples were collected from a small cut in the tail vein and blood glucose levels were measured automatically with the aid of a one touch glucometer (Bionime, Pharmatec, Tunisia).

After one month of oral treatment, the animals were sacrificed by decapitation in order to minimize the handling stress and the trunk blood collected. Liver, kidney and pancreas were rapidly removed and cleaned; all these samples were stored at (−80 °C) until used.

### Biochemical analysis

Plasma triglycerides (TG), total cholesterol (T-Ch) and high density lipoprotein cholesterol (HDL-c) concentrations were analyzed by spectrophotometer using commercial kits (Biolabo, France) on an automatic biochemistry analyzer (BS 300, China) at the biochemical laboratory of Hedi Chaker Hospital of Sfax.

Serum Low Density Lipoprotein cholesterol (LDL-c) concentration was determined according to formula of Shertzer et al. [30] and atherogenic indices (AI) were determined according to the Friedewald eqs. [31]:

$$ \begin{array}{l}\mathrm{AI}=\left(\mathrm{T}\hbox{-} \mathrm{Ch}\hbox{-} \mathrm{HDL}\hbox{-} \mathrm{c}/\mathrm{HDL}\hbox{-} \mathrm{c}\right),\\ {}\mathrm{HTR}\left(\%\right)=\mathrm{HDL}\hbox{-} \mathrm{c}/\mathrm{T}\hbox{-} \mathrm{Ch}\ \mathrm{ratio},\\ {}\mathrm{LDL}\hbox{-} \mathrm{c}=\mathrm{T}\hbox{-} \mathrm{Ch}\hbox{-} \left(\mathrm{T}\mathrm{G}/5\right)\hbox{-} \left(\mathrm{HDL}\hbox{-} \mathrm{c}\right)\end{array} $$

The ratios of LDL-c to HDL-c were also calculated.

The serum of α-amylase, lipase pancreatic, AST, ALT, ALP, creatinine, albumin and urea rates were measured by standardized enzymatic procedures using commercial kits from (Biolabo, France) on an automatic biochemistry analyzer (BS 300, China) at the biochemical laboratory of Hedi Chaker Hospital of Sfax.

### Determination of hematological parameters

Horiba ABX 80 Diagnostics (ABX pentra Montpellier, France) was used for the determination of hematological parameters including red blood cells (RBC) and its related indices following manufacturer’s instruction. These include hemoglobin (Hb), mean corpuscular volume (MCV), mean corpuscular hemoglobin (MCH), mean corpuscular hemoglobin concentration (MCHC), white blood cell (WBC) and platelets.

### Antioxidant defense system assay

The antioxidant activities were measured, after the homogenization of the liver, kidney and pancreas in a phosphate buffer (pH 7.4) and centrifuged at 3500 rpm for 30 min. The quantification of lipid peroxidation (MDA) in the liver, kidney and pancreas of experimental groups rats was evaluated by the method of Draper and Hadley [32] and expressed by nmol MDA/mg protein. The AOPP levels were determined according to the method of Kayali et al. [33] and expressed by nmol/mg protein. CAT activity was measured according to Aebi [34] and expressed as mmoles of H_2_O_2_ consumed/(min_mg protein). The activity of SOD was assayed spectrophotometrically as described by Beyer and Fridovich [35] and expressed as U/mg protein. The GSH levels activity was assayed according to the method of Carlberg and Mannervik [36] and expressed as mmoles GSS/(min_mg protein) and the protein estimation was carried out by means of Lowry et al. [37] method using bovine serum albumin as the standard at 660 nm.

### Histopathological examination

At the end of experimental period, all the experimental groups of rats were dissected after being sacrificed; pieces of pancreas, liver and kidney were fixed in a bouin solution for 24 h and then embedded in paraffin. Sections of 5-μm thickness were stained with hematoxylin-eosin (H&E). The slides were photographed with an Olympus U-TU1X-2 camera connected to an Olympus CX41 microscope (Tokyo, Japan).

### Statistical analysis

All the data were expressed as mean values ± standard deviation (S.D). The level of statistical significance was set at *p* < 0.05. SPSS program was employed for comparisons between all groups. In the case of multiple comparisons, repeated measurements of Analysis of Variance (ANOVA) were performed to compare the mean differences between and within groups, using Tukey tests.

## Results

### In vitro study

#### Phytochemical screening of *Cynara scolymus* leaves extracts

Phytochemicals anaylsis of extracts from leaves of *C. scolymus* revealed the presence of flavonoid, cardiac glycosides, and tannins as shown in Table 1.

**Table 1:** Phytochemical screening of *Cynara scolymus* leaves extracts

Extracts	Cardiac glycosides	Triterpenoids	Saponin	Flavonoids	Alkaloid	Tannins
Hexane	+	+	+	+	−	+
Ethylacetate	+	−		+	+	+
Butanol	−	+	−	+	−	+
Ethanol	+	+	+	+	+	+
Aqueous	−	−	−	+	−	+

Sign (+) indicates present and sign (−) indicates absent

#### Estimation of total phenolic and flavonoid content

Maximum amount of TPC was obtained using 75% of ethanol extract from leaves of *C. scolymus* as the extraction solvent and corresponded to 54.54 ± 1.26 mg GA/g dry extract, followed by ethylacetate, aqueous and butanol extracts. TPC of hexane extract was the lowest in comparison with ethanol extract (30.91 ± 9.36 mg GAE/ g dry extract). Moreover, the ethanol extract exhibited also the highest value of TFC recorded of value 12 ± 0.83 mg CE/ g dry extract (Table 2).

**Table 2:** Total phenolic and flavonoids content of *Cynara scolymus* leaves extracts

Sample	Total phenolic content (mg GAE/g dry extract)	Total flavonoids content (mg CE/g dry extract)
Hexane	39.91 ± 9.36	8.19 ± 0.16
Ethylacetate	53.07 ± 0.47	10.32 ± 0.12
Butanol	41.66 ± 2.23	11.21 ± 0.10
Ethanol	54.54 ± 1.26	12.00 ± 0.83
Aqueous	49.49 ± 0.39	9.49 ± 0.39

Values are expressed as mean S.D. ± (*n* = 3)

#### Antioxidant capacity by method of ABTS•+

As evident from Table 3, The values of antioxidant activity showed that ethanol extract from the leaves of *C. scolymus* was a significantly higher value (499.43 ± 39.72 g Trolox/g dry extract) than in the other extracts, but the lowest level of TEAC was obtained from the hexane extract (104.3 ± 10.73 g Trolox/ g dry extract).

**Table 3:** Antioxidant activities of *Cynara scolymus* leaves extracts

Extracts	ABTS assay (moL Trolox /g dry extract)	Total antioxidant capacities (mg VC /g dry extract)
Hexane	104.3 ± 1.73 ^###^	145 ± 8.67^###^
Ethylacetate	382.60 ± 5.24 ^##^	165.78 ± 9.56^ns^
Butanol	251.93 ± 2.15^###^	109.40 ± 7.15^###^
Ethanol	499.43 ± 3.72^ns^	128.75 ± 8.45^ns^
Aqueous	210.74 ± 8.36 ^###^	140.54 ± 2.45^ns^

Values are given as means ± SD (*n* = 3). ^#^ *p* ≤ 0.05, ^##^ *p* ≤ 0.01, ^###^ *p* ≤ 0.001 were significant compared to ethanol extract from leaves of *C. scolymus*; ^ns^: non-significant changes between all extracts vs ethanol extract from leaves of *C. scolymus*

#### Total antioxidant capacities

Ethanol extract from leaves of *C. scolymus* exhibited the highest value of 128.75 ± 8.45 mg VC /g dry extract of TAC in compared with the others extracts (Table 3).

#### HPLC analysis of *C. scolymus* leaves extract

Accordingly, results obtained, so far, by Folin-ciocalteu needed to be further complemented to qualify and quantify phenolic constitutents in ethanol extract from leaves of *C. scolymus* under investigation.

**Fig. 1:**
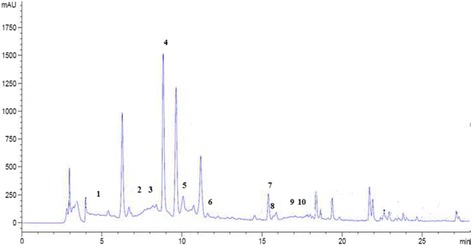
HPLC chromatogram of a standard mixture of polyphenolic compounds. Peaks:1, Hydroxytyrosol; 2, Tyrosol; 3, 4HObenzoic; 4,verbascoside; 5, Apigenin-7-glucoside; 6, Oleuropein; 7,Quercetin; 8, Pinoresinol; 9,cinnamic acid; 10; Apigenin

**Fig. 2:**
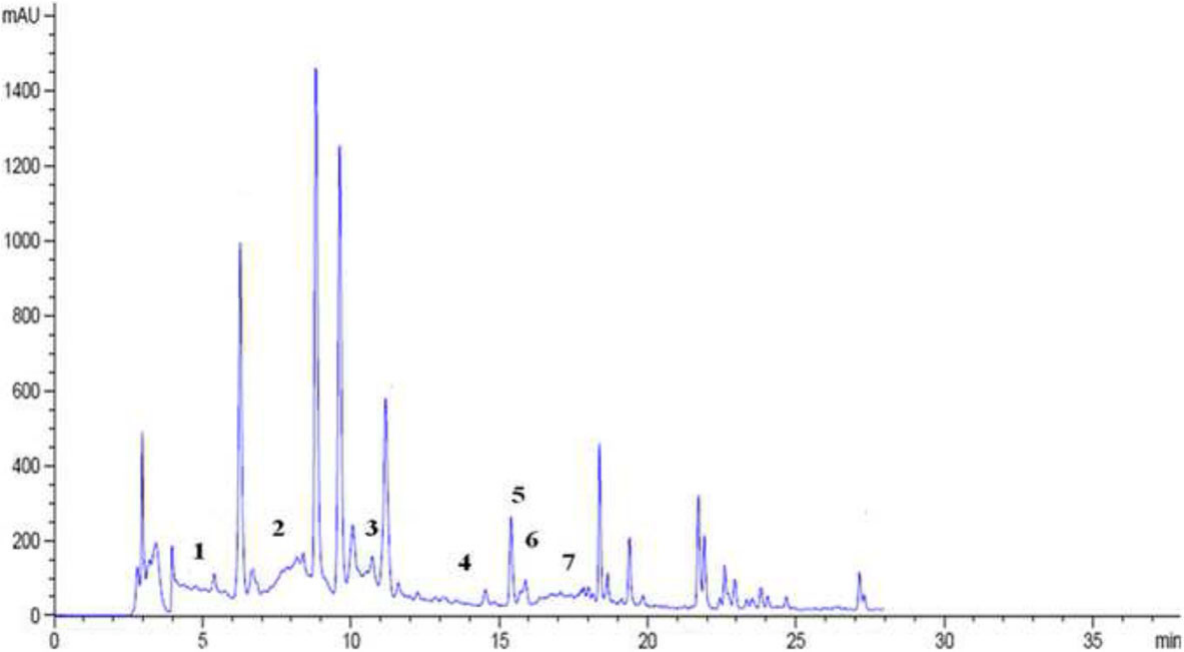
HPLC chromatogram of ethanol extract from leaves of *Cynara scolymus*. Peaks: 1, Hydroxytyrosol; 2, Verbascosie; 3, Apigenin-7-glucoside; 4, Oleuropein; 5, Quercetin; 6, Pinoresinol; 7, Apigenin

The results are expressed as μg/g of content. The findings revealed the presence of seven phenolic compounds namely: Hydroxytyrosol, Verbascoside, Apigenin-7-glucoside, Oleuropein, Quercetin, Pinoresinol and Apigenin. Those compounds were partially identified by the comparison of their retention times and UV spectra to those of authentic standard analyzed under identical conditions (Fig. 1). The results demonstrated that these compounds could be classified as the most common phenols simple was hydroxytyrosol, the most common secoiridoides was oleuropein and verbascoside, the most common lignans was pinoresinol and the most flavonoids were quercetin, apigenin, and apigenin7-Glu (Fig. 2).

HPLC analysis indicated that ethanol extract have significant amount of flavonoid content; Quercetin content (0,64 μg/g), Apigenin content (0,88 μg/g) and Apigenin-7-glucoside content (0,99 μg /g) (Table 4).

**Table 4:** Identification and quantification of phenolic compounds of ethanol extract from leaves of *Cynara scolymus* by using HPLC analysis

Detected compounds	Retention time (min)	Composition (μg/g)
(1) Hydroxytyrosol	5.06	0.28 ± 0.21
(2) Verbascoside	8.82	3.81 ± 0.15
(3) Apigenin −7-glucoside	10.71	0.99 ± 0.13
(4) Oleuropein	11.60	0.58 ± 0.07
(5) Quercetin	15.39	0.64 ± 0.02
(6) Pinoresinol	15.87	0.46 ± 0.01
(7) Apigenin	17.06	0.88 ± 0.1

Values represent means ± S.D. (*n* = 3)

#### α-amylase inhibition activity

The results showed that the ethanol extract from leaves of *C. scolymus* evidenced a better pancreatic α- amylase inhibition with an IC50 value of 72.22 μg/mL as compared to acarbose specific inhibitor (IC50 = 14.83 μg/mL) (Table 5). The potential in vitro inhibitory effect of ethanol extract compared to the other extracts leads us to use it for in vivo investigation.

**Table 5:** In vitro α-amylase inhibition of *Cynara scolymus* leaves extract and acarbose commercial drug

Sample	Concentration (μg/mL)	% Inhibition	IC 50 (μg/mL)
Acarbose	50	31.05 ± 0.08	14.83
	100	67.40 ± 0.59	
	200	90.35 ± 0.31	
Hexane	50	17.46 ± 0.05	-
	100	25.63 ± 0.19	
	200	30.11 ± 0.28	
Ethylacetate	50	29.90 ± 0.86	-
	100	33.79 ± 1.20	
	200	47.50 ± 1.43	
Butanol	50	53.09 ± 0.88	82.19
	100	60.83 ± 1.02	
	200	79.08 ± 0.97	
Ethanol	50	50.18 ± 0.58	72.22
	100	69.23 ± 0.84	
	200	83.71 ± 0.77	
Aqueous	50	46.91 ± 1.27	95.78
	100	52.20 ± 0.99	
	200	66.24 ± 1.13	

Values are given as means ± S.D. (*n* = 3)

### In vivo study

#### Acute toxicity studies

The ethanol extract from leaves of *C. scolymus* did not show any sign of acute toxicity up to a dose of 2000 mg/kg. The behavior of the animals was clearly observed for the first 8 h, then at an interval of every 8 h during next 72 h. The ethanol extract did not produce a significant change in animal behavior or mortality. Subsequent biological evaluation assays were, therefore, performed using the ethanol extract from leaves of *C. scolymus* at concentration of (200 mg/kg,b.w.) and (400 mg/kg,b.w.).

#### Effect of *Cynara scolymus* leaves extract on OGTT

**Fig. 3:**
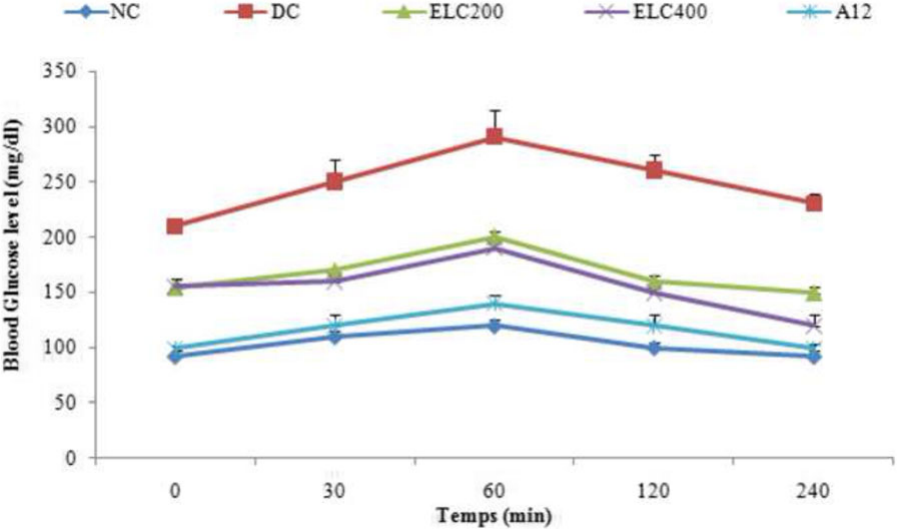
Oral glucose tolerance levels in experimental groups of rats. Normal (NC) and Diabetic control (DC). ELC200&400: Ethanol extract from leaves of *C. scolymus* at doses of 200 and 400 mg/kg per day; A12: Acarbose at dose of 12 mg/kg per day for 28 days. Values are given as means ± SD (*n* = 8 for each group)

The blood glucose levels of control rats were noted to increase to a maximum of 122.3 mg/dL after 60 min of oral glucose challenge and to return to normal levels after 240 min (Fig. 3). The administration of 2 g/mL glucose to untreated diabetic rats induced an increase in their blood glucose levels, with peak of 312.11 mg/dL. The administration of acarbose and ethanol extract at 200 mg/kg and 400 mg/kg doses, on the other hand, decreased significantly (*p* < 0.001) the glucose peak in blood glucose levels to 92, 58, 150, 06 and 116.28 mg/dL, respectively.

These results suggest that increased levels of glucose tolerance may be due to increased secretion of insulin. These results indicate that the ethanol extract of *C. scolymus* possesses a hypoglycemic effect.

#### Effect of *Cynara scolymus* leaves extract on body weight

The result of Table 6 was shown that diabetes induced a significant decrease (*p* < 0.001) in body weight of diabetic rats by 32.96% as compared to the control group. In fact, oral administration of ethanol extract (200-400 mg/kg) induced a significant increase (*p* < 0.001) in body weight by 34.20% and 27.47%, respectively, after 28 days.

**Table 6:** Effect of *Cynara scolymus* leaves extracts on body weight gain of experimental groups of rats

Groups of treatment	Day1	Day 7	Day14	Day 21	Day 28
Cont	180.33 ± 0.57	182.13 ± 1.84	186.00 ± 1.09	197.50 ± 1.05	205.65 ± 4.92
Diab	181.48 ± 1.29^ns^	176.85 ± 2.74^***^	173.22 ± 1.04^***^	166.66 ± 2.08^***^	154.66 ± 5.50^***^
Diab + Ethanol extract of leaves *C. scolymus* (200 mg/kg)	185.00 ± 6.24^ns^	195.39 ± 0.74^###£^	199.41 ± 0.71^###£££^	204.33 ± 1.90^###£££^	207.56 ± 2.44^###£££^
Diab + Ethanol extract of leaves *C. scolymus* (400 mg/kg)	180.75 ± 0.55^£££^	184.82 ± 0.23^###£££^	188.04 ± 0.94^###£££^	192.00 ± 2.64^###^	196.00 ± 1.00^###^
Diab + Acarbose (12 mg/kg)	189.70 ± 0.60^#^	192.76 ± 1.36^###^	193.20 ± 0.82^###^	194.13 ± 2.19^###^	197.16 ± 2.46^###^

Diabetic rats were orally administered with the ethanol extract from leaves of *C. scolymus* and acarbose at the doses mentioned earlier for 28 days. Values are given as means ± SD (*n* = 8). ^***^ *p* ≤ 0.001 were considered significant compared to control group; ^#^ *p* ≤ 0.05, ^###^ *p* ≤ 0.001 were considered significant compared to diabetic rat.; ^£^ *p* ≤ 0.05, ^£££^ *p* ≤ 0.001 compared to diabetic rats treated with acarbose; ^ns^: non-significant changes between diabetic treated groups vs. the control group after 28 days treatment

#### Effect of *Cynara scolymus* leaves extract on blood glucose levels

**Fig. 4:**
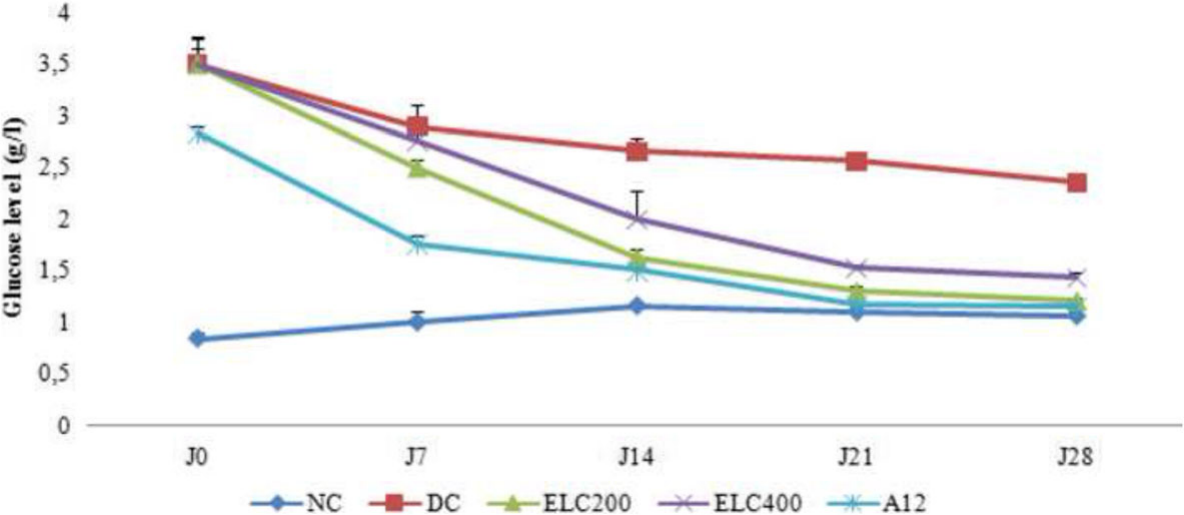
Blood glucose levels in experimental groups of rats. Normal (NC) and Diabetic control (DC). ELC200&400: Ethanol extract from leaves of *C. scolymus* at doses of 200 and 400 mg/kg per day; A12: Acarbose at dose of 12 mg/kg per day for 28 days. The values are means ±SD (*n* = 8 for each group)

The results of blood glucose levels in experimental groups of rats were shown in Fig. 4.

There was a significant increase (*p* < 0.05) of 54.49% in blood glucose level in diabetic rats when compared with normal control rats. Administration of ethanol extract from leaves of *C. scolymus* (200-400 mg/kg) and acarbose to diabetic rats induced a significant decrease of 42.84%, 37.91% and 23.21% in blood glucose level after 28 days of treatment compared to control group. The ethanol extract and acarbose treated groups remained to have higher serum glucose level throughout the experimental period when compared with control groups.

#### Effect of *Cynara scolymus* leaves extract on α-amylase and lipase activity in serum

**Fig. 5:**
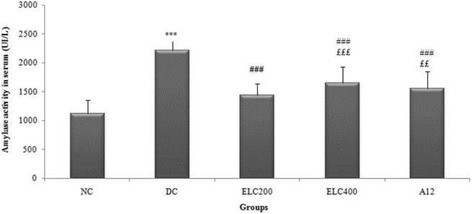
Levels of α-amylase activity in serum of experimental groups of rats. Normal (NC) and Diabetic control (DC). ELC200&400: Ethanol extract from leaves of *C. scolymus* at doses of 200 and 400 mg/kg per day; A12: Acarbose at dose of 12 mg/kg per day for 28 days. The values are means ±SD (*n* = 8 for each group). ^***^ *p* ≤ 0.001 was considered significant compared to control group; ^####^ *p* ≤ 0.001 was considered significant compared to diabetic rat. ^£££^ *p* ≤ 0.001, ^££^ *p* ≤ 0.01 compared to diabetic rats treated with acarbose

**Fig. 6:**
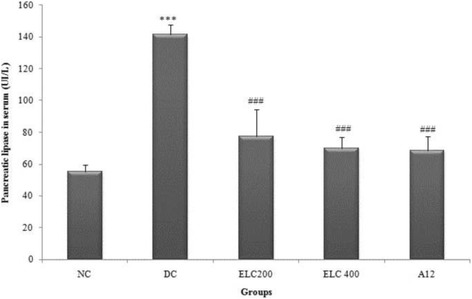
Levels of lipase pancreatic activity in serum of experimental groups of rats. Normal (NC) and Diabetic control (DC). ELC200&400: Ethanol extract from leaves of *C. scolymus* at doses of 200 and 400 mg/kg per day; A12: Acarbose at dose of 12 mg/kg per day for 28 days. The values are means ±SD (*n* = 8 for each group). ^***^ *p* ≤ 0.001 was considered significant compared to control group; ^####^ *p* ≤ 0.001 was considered significant compared to diabetic rats

Figures 5 and 6 evidenced that, compared to control groups, there was a significant rise in the activities of both α-amylase and lipase pancreatic in serum of untreated diabetic rats by 47.74% and 60.71%, respectively, (*p* < 0.05). However, the administration of ethanol extract from leaves of *C. scolymus* (200–400 mg/kg) to surviving diabetic rats induced a considerable reduction in serum α-amylase and lipase activity by 17.90%, 12.66% and by 48.33%, 51.78%, respectively.

#### Effect of *Cynara scolymus* leaves extract on hematological parameters

The findings indicated that, compared to control groups, there was a significant decrease in the levels of RBC, Hb, MCV, MCH and MCHC observed in diabetic rats. However, these hematological markers were considerably increased to near normal values after administration of ethanol extract from leaves of *C. scolymus* (200-400 mg/kg), (*p* < 0.001) (Table 7). Furthermore, the levels of serum WBC and platelets were considerably decreased in diabetic rats as compared to control groups. Meanwhile, these levels were increased after oral administration of ethanol extract as compared to diabetic group.

**Table 7:** Effect of *Cynara scolymus* leaves extracts on hematological parameters of experimental groups of rats

Groups of treatment	Cont	Diab	Diab + Ethanol extract of leaves *C. scolymus* (200 mg/kg)	Diab+ + Ethanol extract of leaves *C. scolymus* (400 mg/kg)	Diab + Acarbose (12 mg/kg)
RBC (×10^6^)	6.88 ± 1.31	5.08 ± 1.80^***^	7.32 ± 0.70^###£^	7.50 ± 0.80^###^	7.66 ± 0.30^###^
Hb (g/dl)	13.00 ± 0.60	9.64 ± 3.37^***^	12.54 ± 0.95^###£££^	13.7 ± 0.89^###^	13.86 ± 0.58^###^
MCV (fl)	60.40 ± 4.30	57.00 ± 2.86^***^	58.40 ± 3.00^###£££^	47.53 ± 0.89^###£^	60.80 ± 2.68^###^
MCH (pg)	20.00 ± 4.47	19.10 ± 1.46^ns^	18.60 ± 1.14^###£^	18.22 ± 0.41^###^	18.00 ± 0.45^###^
MCHC (g/dl)	32.6 ± 5.27	16.87 ± 0.25^***^	28.53 ± 1.37^###£^	29.60 ± 1.14^###£^	30.00 ± 0.71^###^
WBC (×10^3^/ul)	8.22 ± 0.93	5.30 ± 0.70^***^	5.2 ± 0.35^£££^	6.4 ± 0.53^#£££^	8.26 ± 0.34^###^
PLT (×10^3^ /ul)	900 ± 0.63	260 ± 0.34^***^	705 ± 0.45^###£^	605 ± 0.87^###^	640 ± 0.54^###^

Diabetic rats were orally administered with the ethanol extract from leaves of *C. scolymus* and acarbose at the doses mentioned earlier for 28 days. Values are given as means ± SD (*n* = 8). *** *p* ≤ 0.001 were considered significant compared to control group; ^#^ *p* ≤ 0.05, ^###^ *p* ≤ 0.001 were considered significant compared to diabetic rat.; ^£^ *p* ≤ 0.05, ^£££^ *p* ≤ 0.001 compared to diabetic rats treated with acarbose; ^ns^: non-significant changes between diabetic treated groups vs. normal control group after 28 days treatment. *RBC* Red blood cells, *Hb* Hemoglobin, *MCV* Mean corpuscular volume, *MCH* Mean corpuscular hemoglobin, *MCHC* Mean corpuscular concentration, *WBC* White blood cell, *PLT* Platelet count

#### Protective effect of *Cynara scolymus* leaves extract in oxidative stress and hepatic dysfunction parameters of diabetic rats

Our results revealed that hepatic tissues of diabetic rats underwent a decrease in SOD, CAT and GSH activities and an increase in MDA and AOPP levels (Table 10). Furthermore, in diabetic rats, a significant increase of T-Ch, TG and LDL-c levels in serum by 23.62%, 62.45% and 44.88% as well as significant decrease of HDL-c level in serum by 38.63%, were observed as compared to control groups (Table 8). The result showed an increase in the indices of liver dysfunction parameters of diabetic untreated rats such as AST, ALT and ALP activities by 32.63%, 41.36% and 27.01%, as compared to control group (Table 9).

**Table 8:** Effect of *Cynara scolymus* leaves extracts on plasma lipid profile levels of experimental groups of rats

Lipid profile	Control	Diab	Diab + Ethanol extract of leaves *C. scolymus* (200 mg/kg)	Diab + Ethanol extract of leaves *C. scolymus* (400 mg/kg)	Diab + Acarbose (12 mg/kg)
TG (mmol/L)	0.57 ± 0.06	1.51 ± 0.36^***^	0.6 ± 0.05^###^	0.54 ± 0.05^###^	0.64 ± 0.07^###^
T-Ch (mmol/L)	1.45 ± 0.26	1.83 ± 0.11^*^	1.59 ± 0.05^#£££^	1.67 ± 0.06^#£££^	1.28 ± 0.20^###^
LDL-c (mmol/L)	0.49 ± 0.17	0.9 ± 0.08^***^	0.56 ± 0.15^###^	0.6 ± 0.10^###^	0.55 ± 0.21^###^
HDL-c (mmol/L)	0.52 ± 0.07	0.27 ± 0.01^***^	0.47 ± 0.03^###£^	0.50 ± 0.07^###£^	0.33 ± 0.04^###^
HTR (%)	23.05 ± 5.88	15.69 ± 0.50^*^	26.69 ± 1.82^###^	29.53 ± 3.21^###^	28.10 ± 3.27^###^
AI	2.00 ± 0.07	3.83 ± 0.74^***^	2.54 ± 0.20^#^	2.4 ± 0.39^#^	2.58 ± 0.44^#^
LDL-c/HDL-c	1.28 ± 0.15	2.32 ± 0.15^***^	2.11 ± 0.09^#^	1.6 ± 1.10^###^	1.73 ± 0.41^#^

Diabetic rats were orally administered with ethanol extract from leaves of *C. scolymus* and acarbose at the doses mentioned earlier for 28 days. Values are given as means ± SD (*n* = 8). ^*^ *p* ≤ 0.05, ^***^ *p* ≤ 0.001 were considered significant compared to control group; ^#^ *p* ≤ 0.05, ^###^ *p* ≤ 0.001 were considered significant compared to diabetic rat.; ^£^ *p* ≤ 0.05, ^£££^ *p* ≤ 0.001 compared to diabetic rats treated with acarbose, ^ns^: non-significant changes between diabetic treated groups vs. the control group after 28 days treatment. AI = (T-Ch-HDL-c)/HDL-c, HTR (%) = HDL-C/T-Ch ratio

**Table 9:** Effect of *Cynara scolymus* leaves extracts on liver-kidney dysfunction of experimental groups of rats

Groups of treatment	Cont	Diab	Diab + Ethanol extract of leaves *C. scolymus* (200 mg /kg)	Diab + Ethanol extract of leaves *C. scolymus* (400 mg/kg)	Diab + Acarbose (12 mg/kg)
AST (UI/L)	106.6 ± 1.19	158.25 ± 7.67^***^	90.25 ± 2.82^###^	95.14 ± 2.47^###^	101.66 ± 3.17^###^
ALT (UI/L)	35.33 ± 5.68	60.25 ± 8.18^***^	31.00 ± 4.64^###£^	35.16 ± 8.42^###^	37.75 ± 5.73^###^
ALP (UI/L)	150.5 ± 1.57	200.8 ± 2.5^***^	180.00 ± 2.64^###^	186.25 ± 1.81^###^	175.14 ± 1.64^###^
Albumin (g/l)	35.40 ± 1.50	40 ± 0.78^***^	30.57 ± 1.17^#^	32.4 ± 0.46^###^	30.2 ± 0.50^###^
Urea (mmol/L)	4.76 ± 0.57	7.64 ± 0.89^**^	6.41 ± 0.93^###^	4.76 ± 0.57^###£££^	6.98 ± 1.26^ns^
Creatinine (umol/L)	14.64 ± 3.54	26.84 ± 3.77^***^	17.50 ± 3.39^###£^	19.66 ± 1.00^##^	19.51 ± 5.25^###^

Diabetic rats were orally administered with ethanol extract from leaves of *C. scolymus* and acarbose at the doses mentioned earlier for 28 days. Values are given as means ± SD (*n* = 8). ^**^ *p* ≤ 0.01, ^***^ *p* ≤ 0.001 were considered significant compared to control group; ^#^ *p* ≤ 0.05, ^###^ *p* ≤ 0.001 were considered significant compared to diabetic rat.; ^£^ *p* ≤ 0.05, ^£££^ *p* ≤ 0.001 compared to diabetic rats treated with acarbose. ^ns^: non-significant changes between diabetic treated groups vs. the control group after 28 days treatment

**Fig. 7:**
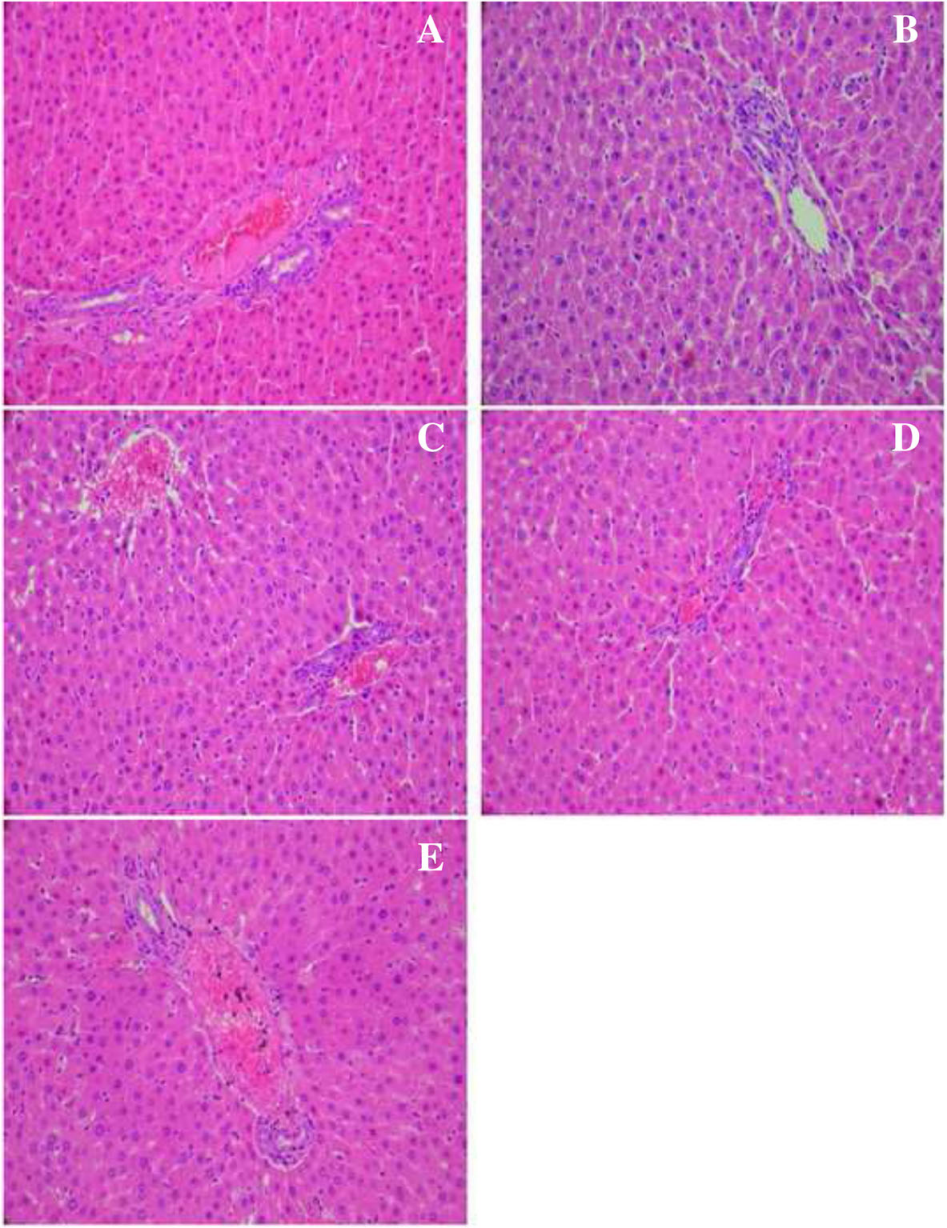
Histopathological studies of liver in experimental groups of rats. **a** Section of the liver from a control rat showing normal architecture; (**b**) alloxan-diabetic rat showing lymphocyte infiltration and a moderate vacuolation of hepatocytes of hepatic cells (**c**-**d**); alloxan-diabetic rat treated with the ethanol extract from leaves of *C. scolymus* at a doses of (200 mg/kg-400 mg/kg) and (**e**); alloxan-diabetic rat treated with acarbose, a protective action was shown (H&E × 400)

The hepatic toxicity leads to problem in metabolism observed by the appearance of vacuolation of hepatic cells with lymphocytic infiltration in diabetic group (Fig. 7b) compared to control group (Fig. 7a).

However, in diabetic rats treated with ethanol extract from leaves of *C. scolymus* at doses (200 mg/kg and 400 mg/kg), a notable protective effect was observed in hepatic function and metabolism (Fig. 7c, d). In fact the administration of ethanol extract increases significantly in CAT, SOD and GSH activities and decreases in MDA and AOPP levels (Table 10) and it was also marked a reduction in lipid profile as evidenced by the significantly decrease observed for ethanol extract (200 mg/kg) of T-Ch, TG and LDL-c levels in serum by 18.11%, 60.47% and 37.77%, respectively, and the increase of HDL-c levels in serum by 15% as compared to diabetic group. Moreover the acarbose group significantly decreased lipid profile in serum as compared to diabetic rats. Moreover, high levels of AI, LDL-c/HDL-c and HTR ratio observed in serum of diabetic rats were significantly reverted towards normal levels after treatment with ethanol extract (200-400 mg/kg) and acarbose group (Table 8), respectively. A concurrent decrease in the indices of hepatic dysfunction parameters (AST, ALT and ALP) was also observed in the plasma of diabetic rats (Table 9). Ethanol extract from leaves of *C. scolymus* and acarbose reduced the appearance of vacuolation of hepatocytes cells with lymphocytic infiltration in diabetic rats as shown in histological results (Fig. 7c–e).

#### Protective effect of *Cynara scolymus* leaves extract in oxidative stress and renal dysfunction markers of diabetic rats

This study showed that the hyperglycemia induced kidney toxicity as indicated by significant decreases in SOD, CAT and GSH activities of diabetic rats by 53.41%, 97.91% and 82.75%, respectively. An increase of 46.45% and 38.61% in MDA and AOPP levels was also observed (Table 10). The results also exhibited an increase in albumin, creatinine, urea contents in plasma by 48.5%, 54.66% and 27.7%, respectively, as compared with the control group (Table 9).

**Fig. 8:**
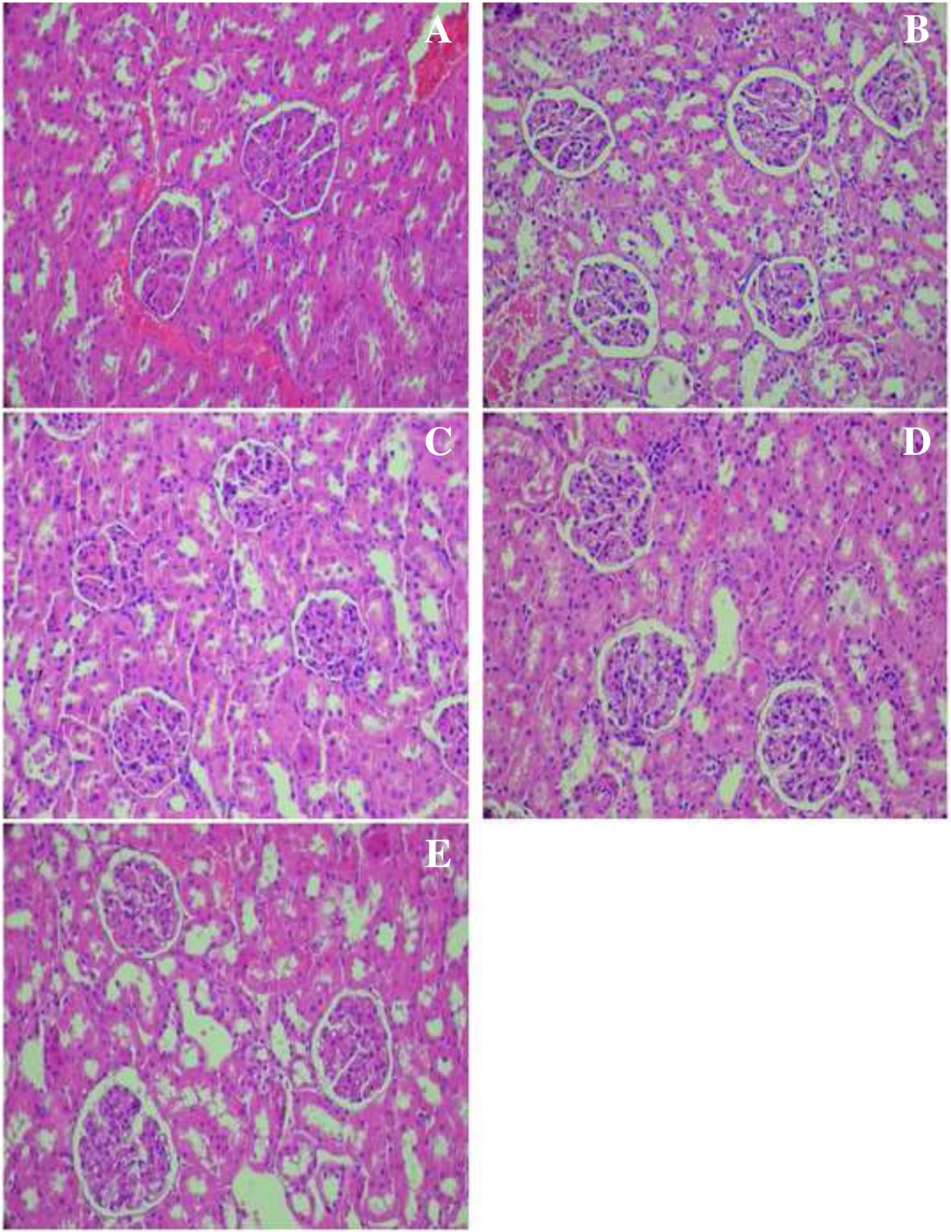
Histopathological studies of kidney in experimental groups of rats. **a** Section of the kidney from a control rats showing normal architecture; (**b**) alloxan-diabetic rat showing Bowman’s space size and glomerular hypertrophy; (**c-d**) alloxan-diabetic rat treated with the ethanol extract from leaves of *C. scolymus* at a doses of (200 mg/kg-400 mg/kg) and (**e**); alloxan-diabetic rat treated with acarbose a potential protective effect was shown (H&E × 400)

In diabetic rats, kidney showed an increase in size of Bowman’s space and glomerular condensation (Fig. 8b) compared with the control group (Fig. 8a). Interestingly, the treatment of ethanol extract from leaves of *C. scolymus* (200–400 mg/kg) and acarbose reverted towards normal levels of the above biochemical parameters and improved all indices related to kidney dysfunction induced by diabetes (Fig. 8c–e).

#### Protective effect of *Cynara scolymus* leaves extract in oxidative stress and pancreas dysfunction parameters of diabetic rats

In diabetic rats, a significant decrease was detected in antioxidant enzymes activities as CAT, SOD and GSH by 12.29%, 98.75% and 93.33%, respectively. An increase of 14.50% in MDA and 43.44% in AOPP levels as compared with the control group was also detected.

**Fig. 9:**
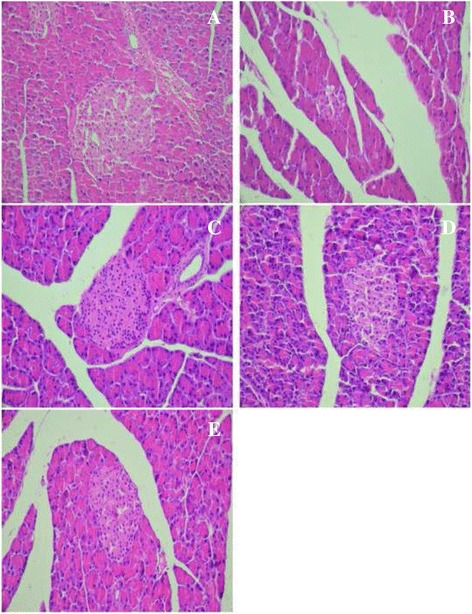
Histopathological studies of pancreas in experimental groups of rats. **a** Section of the pancreas from a control rats showing normal β-cells of pancreas; (**b**) alloxan-diabetic rat a moderate β-cells atrophy; (**c-d**) alloxan-diabetic rat treated with the ethanol extract from leaves of *C. scolymus* at a doses of (200 mg/kg-400 mg/kg) and (**e**); alloxan-diabetic rat treated with acarbose, a partial protective action was shown (H&E × 400)

The administration of ethanol extract from leaves of *C. scolymus* at two doses 200 mg/kg and 400 mg/kg and acarbose was noted to modulate the antioxidant ability of pancreas and to restore the affected values back to normal levels (Table 10). These results were confirmed by histological observation, which revealed a partial protective action of β-cells of pancreas was shown in diabetic treated rats by the ethanol extract (200-400 mg/kg) (Fig. 9c, d). In acarbose treated rats (Diab + Acar), a partial protective action was observed (Fig. 9e).

**Fig. 10:**
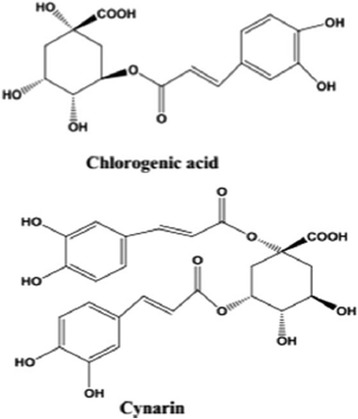
Chemical structure of chlorogenic acid and cynarin by Ben Salem et al. [15]

## Discussion

Alloxan induced diabetic model mimics type 1 is one of the usual substances used for the induction of DM. It caused a reduction in insulin release by a moderate destruction of β- cells of islets of langerhans, thereby, inducing hyperglycemia [38]. Diabetogenic effect of alloxan is characterized by an excess in ROS, leading to toxicity in pancreatic cells which reduces synthesis, release and secretion of insulin [39], while affecting organs such as liver, kidney, and hematopoietic system [40]. Decreased antioxidant enzymes levels and enhanced lipid peroxidation have been well documented in alloxan-induced diabetes [27].

The present work was the first investigation dealing with protective effects of ethanol extract from leaves of *C. scolymus* on diabetes and their complication in liver, pancreas and kidney functions.

The current study revealed that alloxan treatment induced several beta-cell damages, including reduction of degenerative and lytic changes in islet of langerhans of pancreas and insufficiency of insulin secretion. Administration of ethanol extract from leaves of *C. scolymus* at two doses of 200 mg/kg and 400 mg/kg to diabetic rats showed that ethanol extract could be related to partial regeneration or preservation of pancreatic beta cells from progressive damage and to induce more insulin secretion in blood after alloxan treatment. Moreover, alloxan induced diabetic rats exhibited a decline in the body weight. This decrease in surviving rats was related to the structural degradation of proteins due to altered carbohydrate metabolism [41]. However, the treatment of diabetic rats with ethanol extract from leaves of *C. scolymus* increased their body weight which might be due to enhancement metabolism and increased synthesis of structural proteins.

One of the therapeutic approaches for lowering postprondial hyperglycemia is to prevent the absorption of carbohydrate after food intake via inhibition of carbohydrate hydrolyzing enzymes such as key enzyme responsible of the conversion of starch to simple sugars absorbable by small intestine [42, 43]. Therefore, results obtained in vitro revealed that ethanol extract from *C. scolymus* displayed a good concentration-dependent inhibitory effect on α-amylase activity. In fact, α-amylase inhibitory activity of ethanol extract was noted to increase in a dose-dependent way, with the best inhibitory effect of 69.87% being observed at 200 mg/kg of ethanol extract from *C. scolymus*. Moreover, the ethanol extract administration to diabetic rats was noted to inhibit in vivo blood α- amylase, which resulted in a significant decrease in blood glucose by 42.84% and 37.91%. It is worth to note that ethanol extract effect was almost equal to that showed by acarbose.

The potent anti-diabetic effect of this extract might be related to high content of phytochemical compounds, such as terpenoids, phenols, flavonoids, tannins, and cardiac glycosides compounds. In fact, many glycosides have been reported as potential anti-diabetic agents because they exert a good inhibiting action against α- amylase and could have potential in DM prevention as part of dietary strategy [44, 45]. In the other reports, it has been proposed that inhibitory effect of some flavonoids on glucose absorption is due to competitive inhibition of sodium-dependent glucose transporter-1 [45, 46]. Moreover, several studies have reported the antioxidant and anti-radical activities of tannins [15].

These results explained the ability of ethanol extract from leaves of *C. scolymus* to reduce blood glucose levels, which could be attributed (i) to an improvement of peripheral sensitivity to remnant insulin; (ii) inhibition of intestinal dissacharidase activities, such as α- amylase activity and this affected the glucose absorption rate in small intestine as hypoglycemic effects and the strong antioxidant of *C. scolymus* leaves. This provided evidence in favor of the view that ethanol extract could play an important role for the treatment of diabetic patients.

These results corroborated pervious findings which demonstrated that the oral administration of ethanol extract from leaves of *C. scolymus* at doses 200 mg/kg and 400 mg/kg on streptozocotin (STZ) induced diabetic rats exerted a blood glucose lowering [16]. One of the drawbacks of hyperglycemia is the production of ROS which has become the prime cause of chronic complications of diabetes as well as reducing the half-life (t1/2) of RBCs (the normal is 120 days) by disturbing the osmotic fragility of these cells via generating superoxide radicals due to the increase of xanthine oxidase activity; these free radicals are then involved in the oxidation of RBC’s membrane proteins and lipids that make them susceptible to hemolysis [47].

In addition, impaired production of erythropoietin from kidney in diabetic condition alters results in a decrease of RBC count [48].The decrease in RBC count is also the reflection of low Hb content in diabetic patients [49]. Actually, it was reported that some phytochemicals, such as flavonoids, can stimulate the synthesis and secretion of erythropoietin, a glycoprotein hormone, which stimulates stem cells in the bone marrow to produce RBC [50]. On the other hand, increase WBC count observed in diabetic groups which may be due to the pancreatic assault associated with alloxan-induced diabetes [51], while normal levels of WBC indicates the decrease in pancreatic inflammation or necrosis.

In the present study, doses of ethanol extract from leaves of *C. scolymus* (200 mg/kg and 400 mg/kg) significantly improved (*p* < 0.001) the levels of RBC, Hb, MCV, MCH and MCHC in tested groups. MCV is the average of RBC volume used to report of anemia in pure anemic condition or in anemia secondary to other pathological problems like diabetes [52]. MCH and MCHC represent the mean Hb concentration per erythrocyte and per given volume of packed red blood cells, respectively. Both of them also serve as indicators of anemia as they are severely affected by low levels of total Hb in patients [52]. Therefore, the beneficial impact of ethanol extract in improving haematinic profile in alloxan-induced diabetic rats may be due to reducing OS in body, thus, establishing osmotic fragility of RBC and restoring the total Hb concentration in the blood.

The control of postprondial hyperglycemia and the inhibition of OS have often been suggested as important measures in the treatment of DM. It has been hypothesized that one of the principal causes of diabetes-induced injury is the formation of lipid peroxides by free radical derivatives. Hence, the antioxidant activity or the inhibition of generation of free radicals is important in the protection against diabetes-induced damages organs [53].

The body has an effective defense mechanism to prevent and neutralize the free radical-induced damage. This is proficient by a set of antioxidant enzymes such as SOD, CAT and GSH. These enzymes constitute a mutually supportive team of defense against ROS [54, 55]. The present study reported that, in diabetic group, the reduced activities of SOD, CAT and GSH were shown. However, the administration of ethanol extract from leaves of *C. scolymus* (200–400 mg/kg) increases in the level of these enzymes in pancreatic tissues as compared to diabetic group, which indicates the antioxidant activity of ethanol extract.

The level of lipid peroxide is a measure of membrane damage and alterations in the structure and function of cellular membranes. Besides, AOPP is a measure of alteration in the structure of protein. In the present study, the elevation of MDA and AOPP levels in pancreas of diabetic rats was observed. Administration of ethanol extract from leaves of *C. scolymus* significantly reversed these changes. Hence, it is possible that the mechanism of this protection may be due to its antioxidant activity.

Liver and kidney are the most important organs. They play a pivotal role in regulating various physiological processes in the body. Damage to these organs by oxidative stress, was grave consequences in human health [56]. Plasma enzymes including AST and ALT are used in the evaluation of hepatic disorders [57, 58], inflammatory hepatocellular extremely elevated transaminases levels. Serum urea, creatinine level and albumin content are used in the evaluation of renal dysfunction. An augmented level of urea and creatinine cause the osmotic diuresis and depletion of extracellular fluid volume in the diabetic condition in the serum of alloxan-induced diabetic rats [59].

In accordance with these findings, alloxan treatment had a significant role in alteration of liver and renal functions because the activities of AST, ALT and creatinine, serum urea and albumin content were significantly higher than those of the control group’s values [60].

These results were confirmed by histological studies; the hepatic toxicity leads to problem in metabolism observed by a vacuolation of hepatic cells with lymphocytic infiltration in liver. Moreover, hyperglycemia induced kidney toxicity as indicated by an increase in capsular space and glomerular condensation. The administration of ethanol extract from leaves of *C. scolymus* at doses (200 mg/kg & 400 mg/kg) prevents liver and kidney dysfunctions as was observed by low rates in transaminases levels and serum urea, creatinine and albumin content. These positive effects of this extract were confirmed by histological findings. Furthermore, antioxidant activities of ethanol extract prevent oxidative stress as evidenced by the increase of SOD, CAT, GSH and the decrease in AOPP and MDA levels in liver-kidney tissues.

These results were an agreement with the report of Hosseini et al. [61] who showed the hypoglycemic effect of hydroalcoholic extract of *C. scolymus* leaves obtained from Iran region in STZ diabetic rats, whereas it differed from the reports of Heidarian et al. [62] who showed that the aqueous extract of *C. scolymus* obtained from Iran has the beneficial hypoglycemic and hypocholesterolemic effects in diabetic rats, moreover, recently, the studies of Mocelin et al. (2016) demonstrated the potential effect of hypolipidemic and anti-atherogenic effects of aqueous extract from leaves of *C. scolymus* in Brazilian region at doses (150, 300, 600 mg/kg) by decreasing in serum level of T-Ch, LDL accompanied with a decrease in inflammatory markers of IL-1, TNF-α, IFN, CRP, oxidized LDL and anti-oxidized-LDL as compared with hypercholesterolemic rats. These findings are in agreement with the reports of Abdel Magied et al. [63] who showed that the aqueous leaves of *C. scolymus* extracts for the two varieties (Green and violet) from Egypt has a potent hypoglycemic and hypocholesterolemic properties in diabetic rats, these findings could be attributed to the wide range of solubility displayed by the various polar compounds in *C. scolymus* leaves extracts, the degree of polymerization of phenols and their interaction, genetic factors, geographical variations and climatic changes [64]. Besides, protection of liver and kidney dysfunctions by the ethanol extract from leaves of *C. scolymus* inhibited metabolic disorders in diabetic rats as demonstrated by the lowest levels in T-Ch, LDL-C, TG and increase of HDL-C levels in the blood linked to a lower risk of cardiovascular. In addition, AI markedly decreased, causing a significant reduction in LDL-c/HDL-c ratio in diabetic rats. The increase in HDL-c or HTR ratio is one of the most important criteria of anti-atherogenic agents.

Phytochemicals composition showed that the ethanol extract from leaves of *C. scolymus* has been reported to contain bioactive molecules that contribute to antioxidant activity. It include polyphenols, flavonoids, tannins, glycosides and terpenoids, among these major class of phytochemicals found in the plant, flavonoids are naturally occurring phenolic compounds with the strong antioxidant properties because of the presence of aromatic hydroxyl groups [15].

Some flavonoid compounds was identified in ethanol extract from leaves of *C. scolymus* by HPLC,e.g. Quercetin has been reported to reduce the fasting blood glucose and improve glucose tolerance, besides some reporters showed that hypoglycemic effect of quercetin maybe due its antioxidant property, it also protects against oxidative damage and preserve β -cells integrity, several studies reported that quercetin mechanisms of action in diabetes such as decrease lipid peroxidation, increases antioxidant enzymes activity like SOD, GPx and CAT, inhibition of insulin-dependent, activation of phosphoionsinol-3-kinase (PI-3 k) and reduces intestinal glucose concentration in a s study performed by Nuralieve et al. [65] on alloxan induced diabetic rats, in similar study, Vessal et al. [66] has shown that quercetin decreased the plasma glucose level of STZ induced diabetic rats.

Moreover, the ethanol extract from leaves of *C. scolmyus* containing mainly Apigenin-7-glucoside and Apigenin, were also reported for their benefit on treatment and prevention of diabetes and related diseases. Apigenin-7-glucoside and Apigenin reported to inhibit the activities of α-amylase and α-glucosidases.

OGTT was significantly increased with these flavonoid monoglycosides and digylcosides concerning their capacity to lower blood glucose, in addition a significant protective effect over kidney and liver was performed by the aglycones [67].

Many in vitro studies have demonstrated that the antioxidant potential of *C. scolymus* leaves extracts is due to radical scavenging and metal ion chelating effect of its constituents such as cynarin and choloregnic acid [15]. Probably, these compounds through protective effects on β cells reduce the harmful effects of free radical and drug used to induce diabetes, moreover, chlorogenic acid and cynarin increase glucose uptake in peripheral tissues (insulin like activity) and reduce the intestinal absorption of glucose by digestive enzymes inhibiting, repair damaged cells and stimulate the β cells to insulin secretion [68].

Ben Salem et al. [15] revealed that both cynarin and chlorogenic acid are considered to be responsible for its anti-atherogenic actions (Fig. 10), studies showed that the chronic administration of acid chlorogenic improved lipid profiles and skeletal muscle glucose uptake, which in turn improved fasting glucose level, glucose tolerance insulin sensitivity and dyslipidemia in rat model.

**Table 10:** Effect of *Cynara scolymus* leaves extracts on SOD, CAT, GSH, MDA and AOPP levels in liver, pancreas and kidney of experimental groups of rats

Groups	SOD (U/mg protein)	CAT (mmol H_2_O_2_/min/mg protein)	GSH (mg/mg protein	MDA (nmol MDA/mg protein)	AOPP (nmol/mg protein)
Liver					
Control	75.62 ± 7.48	1.79 ± 1.34	33.88 ± 1.00	10.00 ± 5.25	0.20 ± 0.16
Diab	55.21 ± 4.98^*^	0.63 ± 70.20^*^	13.41 ± 0.39^***^	25.76 ± 1.21^*^	0.47 ± 0.05 ^**^
Diab + Ethanol extract of leaves *C. scolymus* (200 mg /kg)	72.14 ± 4.27 ^#£^	1.89 ± 0.56 ^###£^	52.73 ± 9.82^###^	16.29 ± 5.59 ^##££^	0.31 ± 0.17^###£^
Diab + Ethanol extract of leaves *C. scolymus* (400 mg /kg)	64.85 ± 7.39^#£^	2.19 ± 0.20 ^##£^	34.74 ± 2.49^#^	12.74 ± 7.85 ^#£^	0.26 ± 0.25 ^##££^
Diab + Acarbose (12 mg/kg)	91.52 ± 1.58^#^	1.85 ± 1.91^##^	30.57 ± 2.46^##^	11.47 ± 6.63^###^	0.26 ± 0.22 ^##^
Pancreas					
Control	87.71 ± 3.58	1.25 ± 0.44	6.67 ± 1.93	9.21 ± 1.75	0.27 ± 0.14
Diab	29.62 ± 2.23 ^***^	0.11 ± 0.82^***^	2.01 ± 1.02^*^	23.91 ± 7.70^**^	0.63 ± 0.21^*^
Diab + ethanol extract of *C. scolymus* (200 mg /kg)	64.43 ± 5.23 ^##££^	1.16 ± 0.52^#£^	6.65 ± 3.69^#^	11.2 ± 17.26 ^#£^	0.19 ± 0.20 ^#£^
Diab + ethanol extract of *C. scolymus* (200 mg /kg)	43.63 ± 1.06 ^#££^	0.89 ± 0.76 ^##£^	5.96 ± 5.29^#^	17.56 ± 3.73^#£^	0.17 ± 0.15 ^##££^
Diab + Acarbose (12 mg/kg)	55.15 ± 4.92^##^	0.78 ± 0.12 ^###^	2.46 ± 1.23^##^	16.71 ± 1.75^#^	0.31 ± 0.17^#^
Kidney					
Control	46.59 ± 5.10	2.08 ± 0.26	17.25 ± 3.97	21.22 ± 2.23	0.10 ± 0.04
Diab	11.14 ± 3.70 ^*^	0.60 ± 0.36^***^	7.51 ± 0.29^*^	34.75 ± 1.23^**^	0.27 ± 0.04^**^
Diab + ethanol extract of leaves *C. scolymus* (200 mg/kg)	18.23 ± 5.54 ^##££^	2.02 ± 0.57 ^##£^	20.07 ± 1.52^#^	16.03 ± 7.90^##£^	0.11 ± 0.00 ^###££^
Diab + ethanol extract of leaves *C. scolymus* (400 mg/kg)	21.66 ± 1.83^#^	1.28 ± 0.57 ^#£^	23.54 ± 8.72^#^	20.64 ± 1.40^#£££^	0.12 ± 0.01 ^##£^
Diab + Acarbose (12 mg/kg)	28.36 ± 1.67 ^##^	1.96 ± 0.47 ^###^	18.00 ± 0.87^##^	19.31 ± 6.50^###^	0.16 ± 0.02 ^###^

Diabetic rats were orally administered with ethanol extract from leaves of *C. scolymus* and acarbose at the doses mentioned earlier for 28 days. Values are given as means ± SD (*n* = 8). ^*^ *p* ≤ 0.05, ^**^ *p* ≤ 0.01, ^***^ *p* ≤ 0.001 were considered significant compared to control group; ^#^ *p* ≤ 0.05, ^##^ *p* ≤ 0.001, ^###^p ≤ 0.001 were considered significant compared to diabetic rat.; ^£^ *p* ≤ 0.05, ^££^ *p* ≤ 0.01 ^£££^ *p* ≤ 0.001 compared to diabetic rats treated with acarbose

These results support the ethnopharmacological application of whose anti-diabetic effect of ethanol extract from leaves of *C.scolmyus* may be attributed to its flavonoid compounds exert on hyperglycemia and their role in the protection of vital organs that can be damaged by this abnormal status.

Therefore, in the current study the reduction of atherogenic parameter in treated groups with ethanol extract from leaves of *C. scolymus* is due to bioactive flavonoid compounds. These results indicate that ethanol extract has a lipid-lowering effect on the diabetic rats. The mechanism of this hypocholesterolemic action maybe due to the inhibition of dietary cholesterol absorption in the intestine or its production by the liver or stimulation of the biliary secretion of cholesterol and cholesterol excretion in the faeces [42]. These results were confirmed by several previous studies have reported a marked increase in the glycogen content in the liver and soleus muscle and exhibited a significant decreases in glucose level accompanied with a rise in T-Ch rats in treated group with hydro-alcoholic extract of *Soldigo chilensis* (Astreaceae) at doses (125,250 and 500 mg/kg).

## Conclusion

The current study demonstrates that the ethanol extract from leaves of *Cynara scolymus* at 200 and 400 mg/kg doses exhibited anti-hyperglycemic and anti-hyperlipidemic activities through delaying carbohydrates and lipid digestion and absorption, moreover the reversal of altered antioxidant enzymes status and peroxidation damage in tissues by the ethanol extract confirm its antioxidant, anti-peroxidase property and its potential role in defence against free radicals. Therefore, these two doses chosen mentioned the same dose-response of anti-diabetic effect. So, with only two doses of the extract tested, it is difficult to establish a dose-response relationship for this biological effect.

This multiple pharmacological profil might be due to the synergistic effect of its bioactive constituents including particularly, phenolic compounds, Quercetin, Apigenin-7-glucoside, and chloregnic acid. Furthermore, this study provides evidence for the ethnobotanical use of *C. scolymus* leaves in the treatment of diabetes mellitus.

## Abbreviations

ABTS: 2,2-azinobis-3-ethylbenzothiazoline-6-sulfonic acid free radical
AI: Atherogenic Indices
ALP: Phosphatase alkalines
AOPP: Advanced oxidation protein product
AST & ALT: Aspartate and lactate transaminase
b.w: body weight
CAT: Catalase
CE: Catechin equivalent
DM: Diabetes Mellitus
GAE: Gallic acid equivalents
GSH: Glutathion
Hb: Hemoglobin
HDL-C: High Density Lipoprotein Cholesterol
HPLC: High Performance Liquid Chromatography
i.p: intraperitoneal injection
LDL-C: Low Density Lipoprotein Cholesterol
MCH: Mean corpuscular hemoglobin
MCHC: Mean corpuscular concentration
MCV: Mean corpuscular volume
MDA: Malondialdehyde
OGTT: Oral glucose tolerance test
OS: Oxidative stress
RBC: Red blood cells
ROS: Reactive oxygen species
SOD: Superoxide dismutase
STZ: Streptozocotin
TAC: Total Antioxidant Capacities
T-Ch: Total Cholesterol
TFC: Total Flavonoid content
TG: Triglyceride
TG: Triglycerides
TPC: Total Phenolic content
WBC: White blood cell

## References

[1] Zhang BB, Moller DE (2000). New approaches in the treatment of type 2 diabetes. Curr Opin Chem Biol.

[2] Burke JP, Williams K, Narayan KMV (2003). A population perspective on diabetes prevention: whom should we target for preventing weight gain. Diabetes Care.

[3] Wild S, Roglic G, Green A, Sicree R, King H (2004). Global prevalence of diabetes: estimates for the year 2000 and projections for 2030. Diabetes Care.

[4] Vats RK, Kumar V, Kothari A (2005). Emerging targets for diabetes. Curr Sci.

[5] Tagang A, Joseph OA, Alkali K, Olusola OO (2016). Amelioration of Hyperglycaemia, Oxidative Stress and Dyslipidaemia in Alloxan-Induced Diabetic Wistar Rats Treated with Probiotic and Vitamin C. Nutrients.

[6] Sies H (1997). Oxidative stress: oxidants and antioxidants. Exp Physiol.

[7] Mahmoud MS (2015). Protective and therapeutic effectiveness of taurine in diabetes mellitus: a rationale for antioxidant supplementation. Diabetes Metab Syndr Clin Res Rev.

[8] Stamler I, Vaccaro O, Neaton JD, Wentworth D (1993). Diabetes, other risk factors and 12-yrs cardiovascular mortality for men screened in the multiple risk factor intervention trial. Diabetes Care.

[9] Goldstein JL, Schrott HG, Hazzard WR (1973). Hyperlipidemia in coronary heart disease, genetic analysis of lipid levels in 176 families and delineation of a new inherited disorder, combined hyperlipidemia. J Clin Invest.

[10] Kasetti RB, Nabi SA, Swapna S, Apparao C (2012). Cinnamic acid as one of the antidiabetic active principle(s) from the seeds of *Syzygium alternifolium*. Food Chem Toxicol.

[11] Pushparaj P, Tan CH, Tan BKH (2000). Effects of *Averrhoa bilimbi* leaf extract on blood glucose and lipids in streptozocin-diabetic rats. J Ethnopharmacol.

[12] Tahrani AA, Piya MK, Kennedy A, Barnett AH (2010). Glycaemic control in type 2 diabetes: targets and new therapies. Pharmacol Ther.

[13] Lam KS (2007). New aspects of natural products in drug discovery. Trends Microbiol.

[14] Dragan S, Andrica Maria F, Maria-Corina S, Timar R (2014). Polyphenols-rich natural products for treatment of diabetes. Curr Med Chem.

[15] Ben Salem M, Affes H, Ksouda K, Dhouibi R, et al. Pharmacological studies of artichoke leaf extract and their health benefits. Plant Foods Hum Nutr. 2015; doi:10.1007/s11130-015-0503-8. 10.1007/s11130-015-0503-8 26310198

[16] Entaz B, Kazi-Marjahan A, Geum-Hwa L, Hwa-Young L. et al. β-Cell protection and antidiabetic activities of *Crassocephalum crepidioides* (Asteraceae) Benth. S. Moore extract against alloxan-induced oxidative stress via regulation of apoptosis and reactive oxygen species (ROS). BMC Complementary and Alternative Medicine. 2017;17:179. 10.1186/s12906-017-1697-0 PMC5372275 28356096

[17] Gebhardt R. Antioxidative and protective properties of extracts from leaves of the artichoke (*Cynara scolymus* L.) against hydroperoxide-induced oxidative stress in cultured rat hepatocytes. Toxicol. Appl. Pharmacol.1997;144:279–286. 10.1006/taap.1997.8130 9194411

[18] Kraft K (1997). Artichoke leaf extract- recent findings reflecting effects on lipid metabolism, liver and gastrointestinal tracts. Phytomedicine.

[19] Pollock JRA, Stevens R (1965). Dictionary of organic compounds, vol. 5. 4th ed.

[20] Trease GE, Evans WC. Pharmacognosy. 12th ed. Baillier Tindall: ELBS Publications. 1996;p. 344–539.

[21] Fawole OA, Opara UL, Theron KI. Chemical and phytochemical properties and antioxidantactivities of three pomegranate cultivars grown in South Africa. Food Bioprocess Technol. 2011; doi:10.1007/s11947-011-0533-7.

[22] Dewanto V (2002). X Wu, Adom KK, Liu RH. Thermal processing enhances the nutritional value of tomatoes by increasing total antioxidant activity. J Agric Food Chem.

[23] Re R, Pellegrini N, Pannala AA, Yang M, Rice-Evans C (1999). Antioxidant activity applying an improved ABTS radical cation decolorization assay. Free Radic Biol Med.

[24] Prieto P, Pineda M, Aguilar M (1999). Spectrophotometric quantitation of antioxidant capacity through the formation of a phosphomolybdenum complex: specific application to the determination of vitamin E. Anal Biochem.

[25] Nair SS, Kavrekar V, Mishra A (2013). *In vitro* studies on alpha amylase and alpha glucosidase inhibitory activities of selected plant extracts. Euro J Exp Biol.

[26] Khalid GM (2012). Bashir a, Ganai, et al. β-cell protective efficacy, hypoglycemic and hypolipidemic effects of extracts of *Achillea millifolium* in diabetic rats. Chin J Nat Med.

[27] Ben Abdallah KR, Ben Gara A, Jardak N, et al. Inhibitory effects of Cymodocea Nodosa sulphated polysaccharide on α-amylase activity, liver-kidney toxicities and lipid profile disorders in diabetic rats. Arch Physiol Biochem. 2015; doi:10.3109/13813455. 10.3109/13813455.2015.1107588 26599334

[28] Leila Z, Eliandra S, Luisa HC (2007). Effect of crude extract and fractions from *Vitex megapotamica* leaves on hyperglycemia in alloxan-diabetic rats. J Ethnopharmacol.

[29] Ananthan R, Latha M, Ramkumar KM (2004). Modulatory effects of *Gymnema montanum* leaf extract on alloxan-induced oxidative stress in Wistar rats. Nutrition.

[30] Shertzer HG, Woods SE, Krishan M (2011). Dietary whey protein lowers the risk for metabolic disease in mice fed a high-fat diet. J Nutr.

[31] Friedewald WT, Levy RT, Fredrickson DS (1972). Estimation of the concentration of low density lipoprotein cholesterol in plasma without use of the preparative ultracentrifuge. Clin Chem.

[32] Draper HH, Hadley M (1990). Malondialdehyde determination as index of lipid peroxidation. Methods Enzymol.

[33] Kayali R, Cakatay U, Akcay T, Altug T (2006). Effect of alpha-lipoic acid supplementation on markers of protein oxidation in post-mitotic tissues of ageing rat. Cell Biochem Funct.

[34] Aebi H (1984). Methods in enzymology. Catalase in vitro.

[35] Beyer JRWE, Fridovich I (1987). Assaying for superoxide dismutase activity: some large consequences of minor changes in conditions. Anal Biochem.

[36] Carlberg B (1985). Mannervik. Glutathione reductase Methods Enzymol.

[37] Lowry OH, Rosebrough NJ, Farr AL, Randall RJ (1985). Protein measurement with the Folin phenol reagent. J Biol Chem.

[38] Cnop M, Welsh N, Jonas JC (2005). Mechanisms of pancreatic cell death in type 1 and type 2 diabetes. Diabetes.

[39] Sabu MC, Smitha K, Kuttan R (2002). Anti-diabetic activity of green tea polyphenols and their role in reducing oxidative stress in experimental diabetes. J Ethnopharmacol.

[40] Sakurai K, Katoh M, Someno K, Fujimoto Y (2001). Apoptosis and mitochondrial damage in INS-1 cells treated with alloxan. Biol Pharma Bull.

[41] Chen V, Ianuzzo CD (1982). Dosage effect of streptozotocin on rat tissue enzyme activities and glycogen concentration. Can J Physiol Pharmacol.

[42] Hamden K, Keskes H, Belhaj S (2011). Inhibitory potential of omega-3 fatty and fenugreek essential oil on key enzymes of carbohydrate-digestion and hypertension in diabetes rats. Lipids Health Dis.

[43] Hogan S, Zhang L, Li J (2010). Antioxidant rich grape pomace extract suppresses postprandial hyperglycemia in diabetic mice by specifically inhibiting alpha-glucosidase. Nutr Metab.

[44] Pereira DF, Cazarolli LH, Lavado C (2011). Effects of flavonoids on α-glucosidase activity: potential targets for glucose homeostasis. Nutrition.

[45] Sireesha Y, Kasetti RB, Nabi SA, Swapna S, Apparao C (2011). Antihyperglycemic and hypolipidemic activities of *Setaria italica* seeds in STZ diabetic rats. Pathophysiology.

[46] Cazarolli LH, Zanatta L, Alberton EH (2008). Flavonoids: cellular and molecular mechanism of action in glucose homeostasis. Mini-Rev Med Chem.

[47] Saba AB, Oyagbemi AA, Azeez OI (2010). Antidiabetic and haematinic effects of Parquetinanigrescens on alloxan induced type-1 diabetes and normocytic normochromic anaemia in Wistar rats. Afr Health Sci.

[48] Thomas DR (2008). Anemia in diabetic patients. Clin Geriatr Med.

[49] Thomas MC, MacIsaac RJ, Tsalamandris C, Power D, Jerums G (2003). Unrecognized anemia in patients with diabetes: a cross-sectional survey. Diabetes Care.

[50] Ohlsson A, Aher SM. Early erythropoietin for preventing red blood cell transfusion in preterm and/or low birth weight infants. Cochrane Database Syst Rev. 2006; doi:10. 1002/14651858. 10.1002/14651858.CD004863.pub2 16856062

[51] Tanko Y, Mabrouk MA, Adelaiye AB, Fatihu MY, Musa KY (2011). Antidiabetic and some haemotological effects of ethylacetate and n-butanol fractions of *Indigofera pulchra* extract on alloxan- induced diabetic Wistar rats. J Diabet Endocri.

[52] Hoffman R, Benz EJ, Shatill SJ, et al. Hematology: Basic Principles and Practice. 2005. 4 th Edn. Elsevier, New York.

[53] Castro JA, Ferrya GC, Castro CR (1974). Prevention of carbon tetrachloride induced necrosis by inhibitors of drug metabolism. Further studies on the mechanism of their action. Biochem Pharmacol.

[54] Amresh G, Kant R, Zeashan H (2007). Gastroprotective effects of ethanolic extract from *Cissampelos pareira* in experimental animals. J Nat Med.

[55] Amresh G, Singh PN (2007). Antioxidant activity of *Cissampelos pareira* on benzo (a) pyrene induced mucosal injury in mice. Nutr Res.

[56] Dinis-Oliveira RJ, Duarte JA, Remio F (2006). Paraquat poisonings: mechanisms of lung toxicity, clinical features, and treatment. Toxicology.

[57] Achliya GS, Wadodkar SG, Dorle AK (2004). Evaluation of hepatoprotective effect of *Amalkadi Ghrita* against carbon tetrachloride induced hepatic damage in rats. J Ethnopharmacol.

[58] Thabrew MI, Joice PDTM, Rajatissa WA (1987). Comparative study of efficacy of *Pavetta indica* and *Osbeckia octandra* in the treatment of liver dysfunction. Planta Med.

[59] Sheela N, Jose MA, Sathyamurthy D, Kumar BN (2013). Effect of silymarin on streptozotocin-nicotinamide-induced type 2 diabetic nephropathy in rats. Iran J Kidney Dis.

[60] Sheweita SA, El-Gabar MA, Bastawy M (2001). CCl4 induced changes in the activity of phase II drug metabolizing enzyme in the liver of mele rats: role of antioxidants. Toxicology.

[61] Seyed Ebrahim H, Soghra M, Farnaz T (2014). Effect of hydro alcoholic extract of artichoke on diabetes treatment and liver enzymes in diabetic adult male rats. Advanced Herbal Medicine.

[62] Heidarian E, Soofiniya Y (2011). Hypolipidemic and hypoglycemic effects of aerial part of *Cynara scolymus* in streptozotocin-induced diabetic rats. Journal of Medicinal Plants Research.

[63] Mona Mohamed Abdel M, Salah EL Din H, Sahar Mohamed Z, Rania Mohamed EL S. Artichoke (*Cynara scolymus* L.) Leaves and Heads Extracts as Hypoglycemic and Hypocholesterolemic in Rats. Journal of Food and Nutrition Research. 2016;4:1 60–68.

[64] Amal D , Drira M, Sana B, Najla H , Kais M, Adel K, Néji Gh. Assessment of polyphenol composition, antioxidant and antimicrobial properties of various extracts of Date Palm Pollen (DPP) from two Tunisian cultivars. Arabian Journal of Chemistry http://doi.org/10.1016/j.arabjc.2015.

[65] Vessal M, Hemmati M, Vasei M. Antidiabetic effects of quercetin in streptozocin-induced diabetic rats. Comp. Biochem. Physiol. T. 2003;135:3:357–364. 10.1016/s1532-0456(03)00140-6 12927910

[66] Nuraliev Iu N, Avezov GA (1992). The efficacy of quercetin in alloxan diabetes. Eksp Klin Farmakol.

[67] Shobana S, Sreerama YN, Malleshi NG. Composition and enzyme inhibitory properties of finger millet (*Eleusine coracana* L.) seed coat phenolics: mode of inhibition of α-glucosidase and pancreatic amylase. Food Chem. 2009; 115:1268–1273.

[68] Natella F, Scaccini C (2012). Role of coffee in modulation of diabetes risk. Nutr Rev.

